# Clinical Efficacy of Acupuncture on Neoadjuvant Chemotherapy with Capecitabine plus Paclitaxel and Radiotherapy in Progressive Gastric Cancer

**DOI:** 10.1155/2022/6156585

**Published:** 2022-07-12

**Authors:** Xiaomei Miao, Hongying Wu, Yan Liu, Shu Zhang, Chaohui Li, Jie Hao

**Affiliations:** ^1^National Physician Hall, Cangzhou Central Hospital, Cangzhou, China; ^2^Department of Hepatopancreatobiliary Surgery, Cangzhou Central Hospital, Cangzhou, China; ^3^Department of Oncology, Cangzhou Central Hospital, Cangzhou, China

## Abstract

**Objective:**

To assess the clinical efficacy of acupuncture on neoadjuvant chemotherapy with capecitabine plus paclitaxel and radiotherapy in progressive gastric cancer.

**Methods:**

In this randomized, double-blind, controlled clinical trial, 70 patients with advanced gastric cancer receiving radio-chemotherapy between May 2018 and June 2020 were assessed for eligibility in our institution and recruited. They were assigned via the random number table method at a ratio of 1 : 1 to receive either neoadjuvant chemotherapy with capecitabine plus paclitaxel and radiotherapy (control group) or acupuncture on neoadjuvant chemotherapy with capecitabine plus paclitaxel and radiotherapy (intervention group). The outcome measures included symptom mitigation, quality of life, and traditional Chinese medicine (TCM) symptom scores.

**Results:**

The two groups showed similar results in abdominal circumference, intraabdominal pressure, and bowel sounds before treatment (*P* > 0.05). Acupuncture plus conventional treatment was associated with better mitigation on intraabdominal pressure (11.08 ± 1.37 vs. 12.17 ± 2.68) and bowel sounds (4 [3, 4] vs. 3 [3, 4]) versus conventional treatment alone (*P* < 0.05). No statistically significant difference in TCM symptom scores was observed between the two groups before treatment (*P* > 0.05). Acupuncture plus conventional treatment resulted in a lower TCM symptom score (24.63 ± 4.56 points) versus conventional treatment (31.17 ± 4.91 points) (*P* < 0.05). The eligible patients given acupuncture showed significantly higher scores of physical function, role function, emotional function, cognitive function, and social function (81.52 ± 5.37, 88.17 ± 5.17, 85.15 ± 6.71, 78.45 ± 5.85, and 80.98 ± 7.14) versus those without acupuncture (52.98 ± 8.23, 69.87 ± 5.54, 68.24 ± 9.22, 61.34 ± 6.27, and 64.79 ± 6.89) (*P* < 0.05).

**Conclusion:**

Acupuncture is effective in the recovery of acute toxicity after radio-chemotherapy for patients with advanced gastric cancer, which provides a certain reference for clinical treatment and is worthy of application and promotion.

## 1. Introduction

Gastric cancer [1] is a malignant tumor with a high clinical incidence [2]. Statistics show [3] that the incidence of gastric cancer in China was 20.31/100,000 and the mortality rate was 21.26/100,000 in 2015, ranking second in the incidence and mortality of malignant tumors, which poses a serious threat to people's health. Epidemiological statistics [4] revealed that factors such as intensified environmental pollution and changes in diet structure have led to an increase in the incidence of gastric cancer in the young population. The 5-year survival rate of early gastric cancer can reach 90% after radical surgery, while that of progressive gastric cancer remains at only 30%–40% [5]. Due to the insidiousness of early symptoms, the disease may have progressed to an advanced stage by the time of diagnosis, at which local or distant metastases may also exist [6], resulting in the inoperability of the patient [7]. Thus, clinical treatment for advanced gastric cancer usually requires radio-chemotherapy [8]. Radio-chemotherapy is currently one of the most effective means of cancer treatment [9, 10]; however, it is associated with acute toxicity [11]. The treatment of gastric cancer in traditional Chinese medicine (TCM) features well-established theoretical guidance, including disease precautions, disease control, and recurrence prevention. Acupuncture [7] is a key component of TCM, consisting of “needling” and “moxibustion.” Needling refers to the use of needles (usually millineedles) to pierce the patient's skin at a certain angle under the guidance of TCM theory, applying twisting and thrusting techniques to stimulate specific parts of the body (the point of piercing is called the acupoint). Moxibustion is the use of prefabricated moxibustion cones or herbs (mugwort is the most commonly used) to heat and fumigate certain acupoints on the body surface to prevent and treat diseases by thermal stimulation [12, 13]. The effectiveness of acupuncture on the recovery of acute toxicity after radio-chemotherapy has been marginally reported [14]. Patients with gastric cancer undergoing gastric cancer surgery suffer from tissue necrosis, tissue damage, acute infection, and acute blood loss, with increased peripheral white blood cell counts and neutrophils, which reduce the immune activity of lymphocytes to a certain extent. In addition, lymph node dissection during gastric cancer surgery can lead to extensive damage, and the use of anesthetic drugs and the resulting inflammatory stimulation may impair the patient's intestinal motility, which is unfavorable to the patient's recovery. In addition, long-term gastrointestinal suppression may lead to intestinal mucosal lesions, increased intestinal pressure, and intestinal paralysis and even result in damage to the mucosal barrier and intestinal displacement in patients. The massive release of neutrophils and inflammatory factors will lead to a cascade of systemic inflammatory responses and organ dysfunction, which seriously compromises the patient's health.

Accordingly, this study was conducted to evaluate the clinical efficacy of acupuncture on the recovery of acute toxicity after radio-chemotherapy in patients with advanced gastric cancer. The results are as follows.

## 2. Materials and Methods

### 2.1. Baseline Data

**Figure 1:**
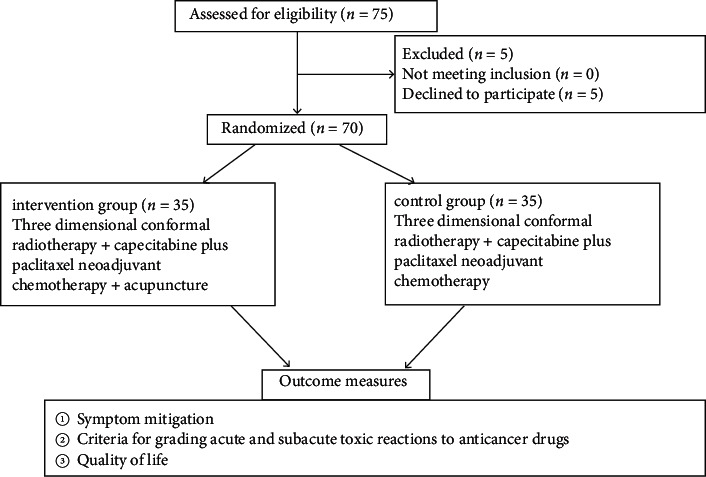
Study flowchart.

In this randomized, double-blind, controlled clinical trial, 70 patients with advanced gastric cancer receiving radio-chemotherapy between May 2018 and June 2020 were assessed for eligibility in our institution and recruited (Figure 1). They were assigned via the random number table method to either a control group (*n* = 35), aged 41–63, or an intervention group (*n* = 35), aged 46–64. This study was certified by the Ethics Committee of the Cangzhou Central Hospital with ethics certificate number 2017-11-14.

### 2.2. Inclusion and Exclusion Criteria

Inclusion criteria were as follows: ① patients who met the diagnosis criteria of gastric cancer in the Diagnosis and Treatment of Common Malignant Tumors in China; ② patients who met the American Joint Committee on Cancer (AJCC) soft tissue tumor staging criteria for gastric cancer stage IIIB and stage IV; and ③ patients with gastrointestinal dysfunction (acute toxicity).

Exclusion criteria were as follows: ① patients with liver and kidney dysfunction; ② patients with cardiopulmonary dysfunction; and ③ patients with neurological dysfunction.

### 2.3. Methods

Patients in the control group were given neoadjuvant chemotherapy with capecitabine plus paclitaxel and radiotherapy. A three-dimensional conformal radiotherapy (3D-CRT) plan was formulated one week before chemotherapy. The details are as follows: first, fix the phantom, and then use the PET/CT simulation positioning machine for positioning. Routine oral administration of 300 ml of water and 20 ml of 76% meglumine was given in the first 20 min before positioning for angiography to display the remnant stomach and small intestine, and the target area and vital organs were delineated layer by layer on a CT image with a layer thickness of 0.5 cm. The clinical target volume (CTV) includes regional lymph nodes, remnant stomach, tumor bed, and other areas. The abovementioned areas are extended by 0.8 cm to be the planned target volume (PTV), which is adjusted according to the individual conditions of the patients (90% isodose PTV). The Pinnacle three-dimensional treatment planning system (TPS) was used to design 3 to 4 irradiation fields. All plans were carried out using the American Varian 2300 C/D high-energy accelerator and conformal lead, and routine bowel surgery was performed before each radiotherapy. The target area of radiotherapy is the gastric lymphatic drainage area, including the left gastric artery, perigastric, splenic hilum, hepatic hilum, peripancreatic lymph nodes, para-aortic lymph nodes, and gastroduodenal ligament. The situation depends on the location of the patient's tumor. If it is the cardia or nearly 1/3 of the lesions, and the accumulated lymph nodes are few, the pancreaticoduodenal ligament and the upper and lower pylorus can be selectively delineated, and the abdominal aorta lymph nodes do not need to be irradiated. For patients with lesions in the gastric antrum, gastric body, and pylorus, the splenic hilar lymph nodes do not need to be irradiated. The parameters are set at a dose of 45 Gy/25 times, 1.8 Gy (1 time/d), 5 times a week. For normal tissue: liver, 60% liver <30 Gy; kidney, at least 2/3 of one kidney <20 Gy; spinal cord, < 45 Gy; heart, 1/3 heart <40 Gy; lung, V20 < 20%, V5 < 50%.

Patients received preoperative adjuvant chemotherapy. Capecitabine (Roche Pharmaceuticals Ltd., Lot No. SH0057) 1 650 mg/(m2-d) was administered orally, d1 to d14, and paclitaxel (Schweppes Ltd., lot 5DO1147) 175 mg/(m2-d) was administered intravenously on d1. The treatment was administered for 2 cycles, with 3 weeks as one cycle. During the chemotherapy period, the three major routine tests, liver and kidney functions, were regularly examined, and nutritional support was enhanced. Gastroscopy and spiral CT scans were performed prior to surgery after chemotherapy to evaluate the effects of chemotherapy. Surgery was usually performed 2–4 weeks after the completion of neoadjuvant chemotherapy. Radical surgery (D2 lymph node dissection) was performed for gastric cancer within 2 weeks of diagnosis.

Patients in the intervention group were given neoadjuvant chemotherapy with capecitabine plus paclitaxel, radiotherapy, and acupuncture. A millineedle of 40 mm or more was used to pierce the following acupoints, including Guanyuan, Qihai, Zusanli, Daheng, Neiguan, Xuehai, Diji, Shuidao, and Guilai, followed by the needling techniques of the lifting-thrusting method and the reinforcing-reducing method. A moxa section of about 2 cm in length was placed on top of the needle handle, about 2-3 cm from the skin, and lit from its lower end, and the skin of the acupoint was covered with kraft paper, with an appropriate warmth felt at the acupoint by the patient. The acupuncture lasted for about 20 min, and the needle was removed after the moxa section was burned out. During the acupuncture, a piece of cardboard could be placed on the acupoint to reduce the heat if the patient felt unbearable heat.

### 2.4. Outcome Measures

① Symptom mitigation: the abdominal circumference, intraabdominal pressure, and bowel sounds of the two groups were recorded in detail and compared.
② Criteria for grading acute and subacute toxic reactions to anticancer drugs [15]: patients' symptoms such as diarrhea, pain, dyspnea, nausea, and vomiting were scored as per the efficacy assessment criteria for acute and subacute toxic reactions to anticancer drugs, with scores ranging from 1 to 4 points. Lower scores indicated milder symptoms. The symptom scores before and after treatment between the two groups were observed and compared.
③ Quality of life: the quality of life of patients was investigated by using the posttreatment follow-up questionnaire, which included five domains: physical function, role function, emotional function, cognitive function, and social function. Each domain has 10 questions, each with 10 points out of 100 points, and the higher the overall score, the better the quality of life.

### 2.5. Statistical Analysis

SPSS 22.0 was used for data analyses. The measurement data were expressed as ($\bar{x}$ ± *s*) and processed using the *t*-test. The count data were expressed as the number of cases (rate) and analyzed using the chi-square test. Differences were considered statistically significant at *P* < 0.05.

## 3. Results

### 3.1. Baseline Data

The baseline clinical characteristics of the intervention group were comparable with those of the control group (*P* > 0.05) (Table 1).

### 3.2. Symptom Mitigation

The two groups showed similar results in abdominal circumference, intraabdominal pressure, and bowel sounds before treatment (*P* > 0.05). Acupuncture plus conventional treatment was associated with better mitigation of intraabdominal pressure (11.08 ± 1.37 vs. 12.17 ± 2.68) and bowel sounds (4 [3, 4] vs. 3 [3, 4]) versus conventional treatment alone (*P* < 0.05) (Table 2).

### 3.3. TCM Symptom Scores

No statistically significant difference in TCM symptom scores was observed between the two groups before treatment (*P* > 0.05). Acupuncture plus conventional treatment resulted in a lower TCM symptom score (24.63 ± 4.56 points) versus conventional treatment (31.17 ± 4.91 points) (*P* < 0.05) (Table 3).

### 3.4. Quality of Life

The eligible patients given acupuncture showed significantly higher scores of physical function, role function, emotional function, cognitive function, and social function (81.52 ± 5.37, 88.17 ± 5.17, 85.15 ± 6.71, 78.45 ± 5.85, and 80.98 ± 7.14) versus those without acupuncture (52.98 ± 8.23, 69.87 ± 5.54, 68.24 ± 9.22, 61.34 ± 6.27, and 64.79 ± 6.89) (*P* < 0.05) (Table 4).

## 4. Discussion

Gastric cancer is a malignant tumor [15], and its incidence and mortality rate in China are far higher than the world average. Patients with advanced gastric cancer are considered ineligible for surgery and therefore require radio-chemotherapy [16, 17]. However, radio-chemotherapy is frequently associated with acute toxicity [11, 18]. In TCM, the development of gastric cancer is attributed to the deficiency of positive Qi and the invasion of evil Qi, and the treatment of gastric cancer is guided by a comprehensive theory including disease precaution, disease control, and recurrence prevention. A recent study has shown the benefits of acupuncture for acute toxicity after radio-chemotherapy in patients with gastric cancer [19]. In TCM, acupuncture is a medical practice that involves the external treatment of internal diseases [20].

In the present study, acupuncture plus conventional treatment was associated with better mitigation on intraabdominal pressure (11.08 ± 1.37 vs. 12.17 ± 2.68) and bowel sounds (4 [3, 4] vs. 3 [3, 4]) versus conventional treatment alone, indicating that acupuncture boosts the patient's intestinal motility and improves the gastrointestinal condition to restore gastric function. The reason could be that the needling on the acupoints of the spleen meridian, stomach meridian, large intestine meridian, and small intestine meridian can ameliorate the malfunction of the human digestive system and that acupuncture features a wide range of indications and provides significant and rapid treatment benefits in long-term medical practice. In addition, acupuncture plus conventional treatment resulted in a lower TCM symptom score (24.63 ± 4.56 points) versus conventional treatment (31.17 ± 4.91 points), which may be attributed to the effective treatment with a high safety profile by acupuncture, as it regulates Qi and blood, restores the relative balance of yin and yang, and harmonizes the functions of the internal organs. Moreover, the eligible patients given acupuncture herein showed significantly higher scores of physical function, role function, emotional function, cognitive function, and social function (81.52 ± 5.37, 88.17 ± 5.17, 85.15 ± 6.71, 78.45 ± 5.85, and 80.98 ± 7.14) versus those without acupuncture (52.98 ± 8.23, 69.87 ± 5.54, 68.24 ± 9.22, 61.34 ± 6.27, and 64.79 ± 6.89). In TCM, disease development and recurrence is essentially the process of the struggle between positive Qi and evil Qi. The main pathogenesis of gastric cancer is a chronic disease of the spleen and stomach, insufficient source of Qi and blood, deficient yin to moisten the stomach, and obstruction by dampness and blood stasis. In the present study, acupuncture was performed at Guanyuan, Qihai, Zusanli, Daheng, Neiguan, Xuehai, Diji, Shuidao, and Guilai acupoints to unblock local Qi and blood, harmonize Yin and Yang of the internal organs, and dispel evil Qi, thereby enhancing patients' quality of life. The limitations of this study lie in the absence of long-term follow-up and the lack of statistics on the long-term disease-free survival time of patients to better determine the long-term efficacy, which will be further investigated in future studies.

## 5. Conclusion

Acupuncture is effective in the recovery of acute toxicity after radio-chemotherapy for patients with advanced gastric cancer, which provides a certain reference for clinical treatment and is worthy of application and promotion.

## Figures and Tables

**Table 1:** Comparison of baseline data ($\bar{x}$ ± *s*).

Groups	*n*	Gender		Age		Clinical stages	
		Male	Female	Range	Mean age	IIIA	IIIB
Intervention group	35	17	18	46–64	52.17 ± 4.38	22	13
Control group	35	20	15	41–63	52.34 ± 5.14	24	11
*t* value	—	0.150					
*P* value	—	0.881					

**Table 2:** Comparison of symptom mitigation ($\bar{x}$ ± *s*$\bar{\text{x}}$).

Groups	*n*	Abdominal circumference (cm)		Intraabdominal pressure (mmHg)		Bowel sounds (*M* [*Q*1, *Q*3], time)	
		Before treatment	After treatment	Before treatment	After treatment	Before treatment	After treatment
Intervention group	35	107.51 ± 10.01	104.08 ± 9.27	15.24 ± 1.65	11.08 ± 1.37^*∗*^	1 (1, 2)	4 (3, 4)^*∗*^
Control group	35	108.52 ± 9.13	106.35 ± 8.63	15.36 ± 1.49	12.17 ± 2.68^*∗*^	1 (1, 2)	3 (3, 4)^*∗*^
*t* value	—	0.438	1.061	0.319	2.146	—	—
*P* value	—	0.663	0.293	0.751	0.035	>0.05	<0.01

Note: ^*∗*^ indicates a statistically significant difference (*P* < 0.05) between pretreatment and posttreatment in the same group.

**Table 3:** Comparison of TCM symptoms scores ($\bar{x}$ ± *s*).

Groups	*n*	Before treatment	After treatment
Intervention group	35	34.11 ± 5.39	24.63 ± 4.56^*∗*^
Control group	35	34.06 ± 5.67	31.17 ± 4.91^*∗*^
*t* value	—	0.043	5.779
*P* value	—	0.966	<0.001

Note: ^*∗*^ indicates a statistically significant difference (*P* < 0.05) between pretreatment and posttreatment in the same group.

**Table 4:** Comparison of quality of life ($\bar{x}$ ± *s*).

Groups	*n*	Physical function	Role function	Emotional function	Cognitive function	Social function
Intervention group	35	81.52 ± 5.37	88.17 ± 5.17	85.15 ± 6.71	78.45 ± 5.85	80.98 ± 7.14
Control group	35	52.98 ± 8.23	69.87 ± 5.54	68.24 ± 9.22	61.34 ± 6.27	64.79 ± 6.89
*t* value	—	17.169	14.285	8.769	11.804	9.658
*P* value	—	<0.001	<0.001	<0.001	<0.001	<0.001

## Data Availability

The datasets used during the present study are available from the corresponding author upon reasonable request.
